# Feasibility of a normoxic N‐vinylpyrrolidone‐based polymer gel (VIPET) dosimeter for three‐dimensional proton beam measurements

**DOI:** 10.1002/acm2.70165

**Published:** 2025-07-14

**Authors:** Ai Nakaoka, Kenji Hotta, Taeko Matsuura, Yoshihiko Hoshino, Hidenobu Tachibana

**Affiliations:** ^1^ Radiation Safety and Quality Assurance Division National Cancer Center Hospital East Chiba Japan; ^2^ Division of Applied Quantum Science and Engineering Faculty of Engineering Hokkaido University Hokkaido Japan; ^3^ Department of Radiology Gunma University Hospital Gunma Japan

**Keywords:** polymer gel dosimeter, proton beam, VIPET

## Abstract

**Background:**

Gel dosimeters enable three‐dimensional dose measurement in x‐ray and charged‐particle therapies. A normoxic *N*‐vinylpyrrolidone‐based polymer gel (VIPET) dosimeter is expected to provide high‐precision proton dose measurements. However, reports on the fundamental performance of VIPET gel dosimeters in proton beam measurement are limited, and the accuracy of position and dose measurements still needs to be determined.

**Purpose:**

We evaluated the accuracy of the VIPET gel dosimeter in proton beam measurement.

**Methods:**

Proton beams of 190 MeV were delivered at dose rates of 2 and 8 Gy/min, and *N*‐vinylpyrrolidone‐based polymer gel dosimeters containing an inorganic salt as a sensitizer (iVIPET) were irradiated with a 10 × 10 cm^2^ field and doses of up to 30 Gy. Magnetic resonance imaging was used for imaging. Key parameters assessed included *R* *2* ‐ dose linearity, dose uncertainty and resolution, dose reproducibility, energy (linear energy transfer [LET]) dependence, dose rate dependence, dose uniformity, and stopping power ratio (SPR).

**Results:**

A strong linear relationship was observed in the 0–30 Gy range. Dose uncertainties were in the range 1%–3%. Three sets of percentage depth dose measurements showed good agreement within 1.6%. Energy (LET) dependence led to measured peak doses being 23% lower than the treatment planning system (TPS) calculations. Proton energy effects in the plateau region were limited and dose rate effects were not recognized. Beam profiles in the axes parallel and perpendicular to the beam axis showed variations of approximately 1.2% and 1.3%, respectively, compared with the TPS calculations. The SPR was consistent with the TPS derived from CT values (1.03).

**Conclusions:**

The VIPET dosimeter showed high reproducibility and uniformity in range and dose measurements of proton therapy beams without energy dependency in the plateau region or dose rate dependency in 2–8 Gy/min. The dosimeter also showed energy (LET) dependence comparable with that reported in previous studies.

## 1 INTRODUCTION

In proton therapy, treatment is performed utilizing the Bragg peak, where energy deposition is maximized near the end of the particle range. This allows for improved tumor control while minimizing the dose to normal tissues, and the number of patients receiving proton therapy is now increasing because of this high tumor control. However, because the dose changes rapidly around the target tumor, high‐precision dose measurement and positioning accuracy are required for quality control in proton therapy.

Gel dosimeters are the only dosimeter tool capable of performing direct three‐dimensional dose measurement. Two main types of gel dosimeters are used for particle beams: polymer and Fricke types. There have been a number of reports on particle beam measurements made using polymer gel dosimeters, including those based on *N*‐vinylpyrrolidone‐argon (VIPAR), bis‐acrylamide nitrogen gelatin (BANG), and *N*‐isopropyl acrylamide (NIPAM), with these having different compositions of ingredients.^1^, ^2^, ^3^

The fundamental characteristics of gel dosimeters for measuring particle beams, such as those using protons or carbon ions, have been investigated. For example, Fricke gel was evaluated with respect to dose linearity, profile, and linear energy transfer (LET) dependence for carbon ions,^4^, ^5^, ^6^, ^7^ and NIPAM was studied for its dose linearity, temperature dependence, dose rate dependence, energy dependence, and LET dependence.^8^ For BANG gel, detailed characteristics related to dose linearity, temperature dependence, dose rate dependence, LET dependence, and profile for protons have been reported.^9^, ^10^ It is general practice that the fundamental characteristics of a newly introduced dosimeter are thoroughly examined before its use. For example, in proton beam measurement using a thermoluminescent sheet‐type 2D dosimeter, Kato and colleagues examined measurement accuracy by evaluating dose reproducibility, dose linearity, dose uniformity, energy dependence, and water‐equivalent thickness measurements.^11^

In contrast, VIPER‐based gel, which has a lower dose rate dependence than methacrylic acid, gelatin, and tetrakis (hydroxymethyl) phosphonium chloride gel, and allows for higher‐precision measurements at lower doses compared with Fricke gel, has only been examined with respect to its range and LET dependence.^12^ Although the VIPET dosimeter belonging to the VIPER family has a low dose‐rate dependency and has been applied in brachytherapy,^13^, ^14^ detailed evaluations need to be performed and published on the fundamental characteristics of proton beam measurements made using it.

The present study, therefore, investigated the fundamental characteristics of proton beam measurement using the VIPET dosimeter.

## 2 METHODS

### 2.1 Gel preparation

In this study, a normoxic *N*‐vinylpyrrolidone‐based polymer gel (VIPET) dosimeter containing an inorganic salt as a sensitizer (iVIPET) was evaluated.^15^, ^16^ We collaborated with Triangle Products Co., Ltd. (Kashiwa, Japan) to manufacture the gel dosimeters. The VIPET gel was composed of 4 wt% *N*‐vinylpyrrolidone (VIP; Wako Pure Chemicals, Japan), 4 wt% *N*, *N’*‐methylenebisacrylamide (Bis; Sigma–Aldrich, UK), 7 wt% gelatin (G2500; Sigma–Aldrich), 85 wt% ultra‐pure water (Direct‐Q; Merck, Germany), 5 mM tetrakis (hydroxymethyl) phosphonium chloride (THPC; Sigma–Aldrich), and 0.2 M magnesium chloride hexahydrate (Wako Pure Chemicals). Each gel was prepared in a beaker under normal atmospheric conditions. First, the gelatin was added to the water and the solution was heated to 55°C and mixed using a magnetic stirrer until the gelatin was completely dissolved. Subsequently, the VIP, Bis, magnesium chloride, and THPC were added sequentially. Finally, glass vials (height, 12 cm; diameter, 4 cm) and a cubic container (12 cm × 12 cm × 12 cm) were filled with the iVIPET gel.

The glass vials were used to evaluate the dose linearity, dose uncertainty, dose resolution, reproducibility of the dose distribution, energy dependence (LET dependence), dose rate dependence, and stopping power ratio. A cubic container of rigid polyvinyl chloride was used to assess the beam profile. For this comparison, one batch of the dosimeter gel was used.

### 2.2 CT, treatment planning, and irradiation

#### 2.2.1 CT image acquisition

Each vial was inserted into a water‐equivalent cuboid phantom (Tough Water Phantom, Kyoto Kagaku, Japan; dimensions, 9.5 × 10 × 14.5 cm^3^). CT images of the cuboid phantom and the cubic container with the gel dosimeters were obtained using an Acquilion ONE scanner (Cannon Medical Systems, Otawara, Tochigi, Japan) with the following image acquisition and reconstruction parameters: tube voltage, 120 kV; tube current, 400 mA; resolution, 0.63 mm/pixel; slice thickness, 1.0 mm.

#### 2.2.2 Treatment planning and irradiation

This study used a proton therapy system (Sumitomo Heavy Industries Ltd. Tokyo, Japan) and an in‐house proton treatment planning system (TPS), with both systems being commissioned and in clinical use since 2018.^17^ The proton dose calculations were made using a simplified Monte Carlo algorithm.^17^

In the cuboid phantom, the cylindrical axis of the vial was aligned with the beam axis. The distance between the surface of the cuboid phantom and the zero point in the gel dosimeter corresponded to a water‐equivalent depth of 16 mm from the surface of the phantom.

**FIGURE 1:**
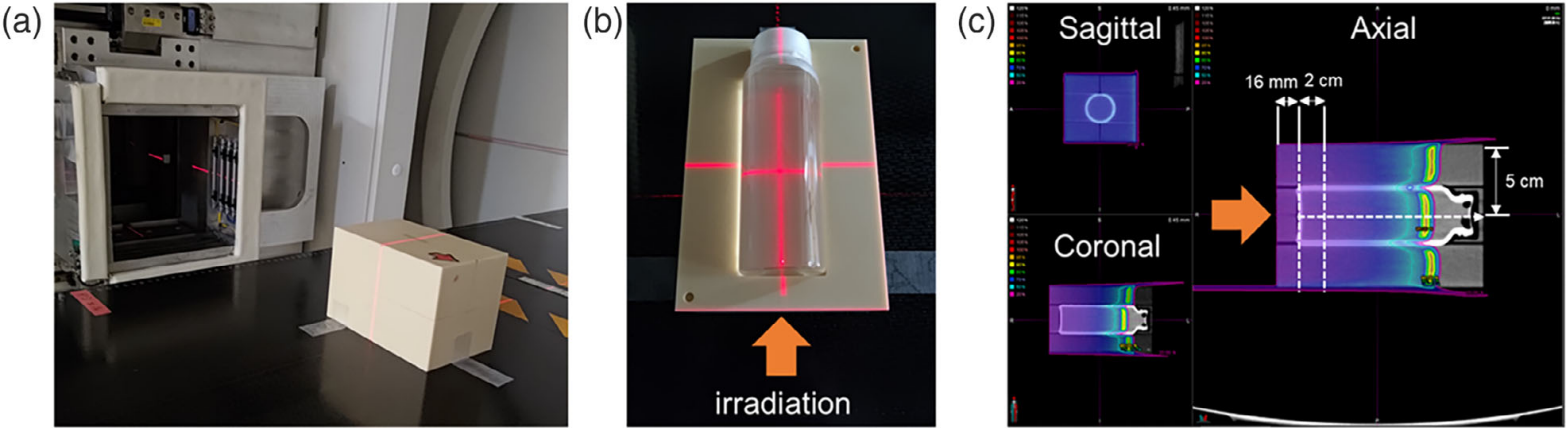
Experimental setup for irradiation of the gel dosimeter. (a) The setup. (b) The center of the gel dosimeter was set corresponding to the isocenter and was irradiated along the arrow direction. The calculated dose distribution of the vial is shown in (c). The vial was placed at a depth of 5 cm in a water‐equivalent cuboid phantom. The parameters were: field size 10 ×10 cm^2^, 5 Gy irradiation, 190 MeV at a 2 cm depth in the vials.

The field size was 10 × 10 cm^2^, with passive proton irradiation used for all measurements. A range shifter of 65‐mm water equivalent thickness was used to adjust the range. The 190 MeV value is the nominal value and is the energy at the exit of the energy selection system. The energy at the nozzle exit is equivalent to about 160 MeV because of energy loss due to scatterers and monitors on the beamline. The experimental setup for the vial irradiation is shown in Figure 1. A mono‐energy 190‐MeV beam with four different target doses (5, 10, 20, and 30 Gy) at 2 cm depth in the vials was set to explore the dose linearity.

For the other tests, 5 Gy was set at 2 cm depth in the vials for all irradiations to the vials. The dose distribution of the vials is shown in Figure 1c. The ranges were measured three times with a mono‐energy 190‐MeV beam to evaluate the reproducibility of the dose distribution. The percentage depth dose (PDD) irradiated with mono‐energy of 190 MeV was compared with the TPS‐calculated PDD to evaluate the energy dependence. In addition, R2 values (as defined in Equation 1) at 10 mm depth intervals from 10–90 mm were evaluated for 190‐MeV beams using irradiation of 5 Gy. The mean R2 value was measured within 2 mm around the central axis at each depth. Subsequently, the residual energies of the protons were calculated using Bethe‐Bloch's theory with the residual range (the distance from the evaluated depth to the peak).^18^ Two dose rates of 2 and 8 Gy/min were set to evaluate dose rate dependence.

**FIGURE 2:**
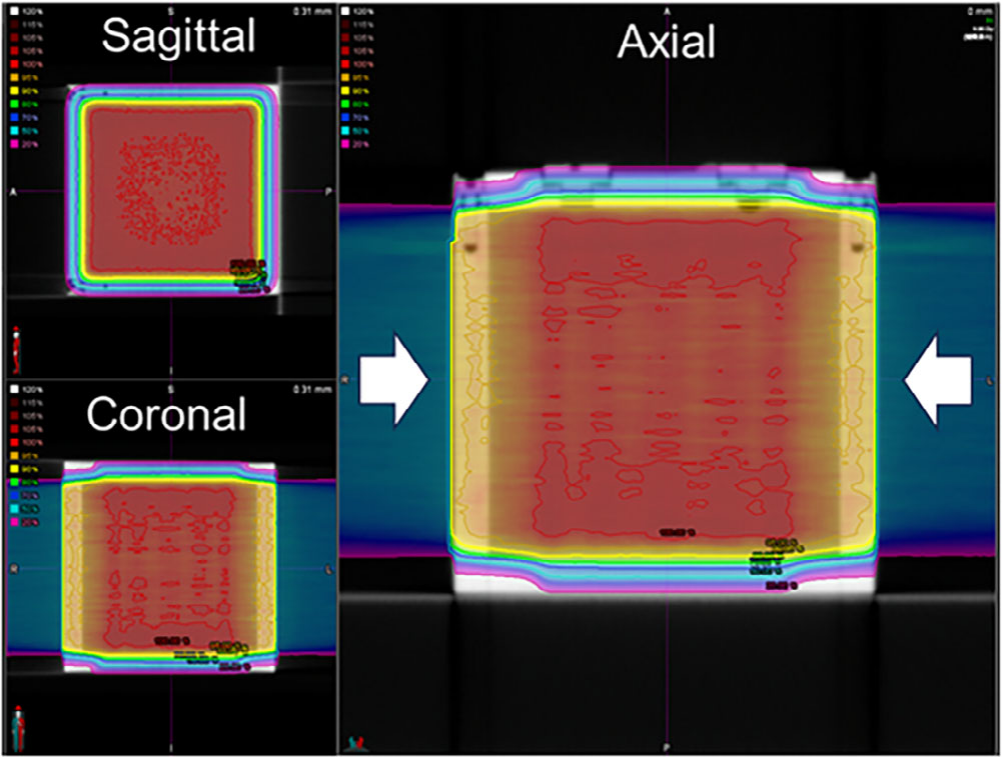
Calculated dose distribution for the beam profile test in the cubic container. White arrows show proton beam direction. The parameters were: field size 10 ×10 cm^2^, target dose of 10 Gy (5 Gy with each SOBP irradiation) set at the isocenter. SOBP, spread‐out Bragg peak.

The 10 × 10 × 10 cm^3^ volume was irradiated uniformly with opposed spread‐out Bragg peak (SOBP) beams to evaluate the beam profile (Figure 2). The total target dose of 10 Gy (5 Gy with each SOBP irradiation) was set at the isocenter.

Polyethylene boards of 2.5 or 5.0 cm thickness were put in front of the vial to evaluate the range shift. The stopping power ratio (SPR) was calculated as:

$$\text{SPR}=\frac{T_{\mathrm{Poly}}}{D_{\mathrm{shift}}}\;\;\times \;WER_{\mathrm{Poly}}$$

where $T_{\mathrm{Poly}}$ is the polyethylene thickness, $D_{\mathrm{shift}}$ is the measured range shift, and $WER_{\mathrm{Poly}}$ is the water‐equivalent ratio of polyethylene. In this study, two range shifts were calculated as the distances of the peak locations of 2.5 and 5.0 cm for the polyethylene plate against the condition with no polyethylene plate. $WER_{\mathrm{Poly}}$ was set to 1.03.

### 2.3 Magnetic resonance imaging

**FIGURE 3:**
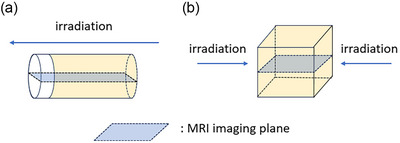
MR imaging planes of (a) the glass vials, and (b) the cubic phantom filled with iVIPET gel. The glass vials and cubic phantom were imaged parallel to the beam axis direction.

More than 24 h after irradiation, the gel dosimeters were sent to an external magnetic resonance imaging (MRI) facility where they were stored at the ambient temperature of the room housing the scanner. All MR images were acquired using a 1.5 T scanner. The sequences for imaging the gel dosimeter were determined before this study.^12^ In this study, we used only two echo times (TE_1_ and TE_2_) because of the limitations of the MRI scanner, as shown in Table 1. Figure 3 shows the scanned plane for the vial and cubic containers.

**TABLE 1:** Magnetic resonance imaging system and acquisition parameters.

Manufacturer	Siemens Healthineers
Model name	Avanto
Coil	Head MATRIX A Tim Coil
Sequence	Spin‐echo
Repetition time	4000 ms
Echo time	12/250 ms
Number of excitations	4
Field of view	256 mm × 256 mm
Pixel size	1 mm × 1 mm
Data depth	16 bits
Pixel band width	130 Hz
Slice Thickness	3 mm

### 2.4 MRI analysis of the gel dosimeters

The reproducibility of the dose distribution, dose linearity, LET dependence, energy dependence, dose rate dependence, and stopping power ratio were evaluated from the MRI of the vials and cubic containers with the dosimeter gel. $T_{2}$ refers to the spin‐spin relaxation time, and R2 is the corresponding spin‐spin relaxation rate, meaning it is simply the inverse of $T_{2}$. The dose‐response was assessed by analyzing the R2 images, which were calculated from TE_1_‐ and TE_2_‐weighted MR images using Simple IMRT Analysis (V2.8.8; Triangle Products Co., Ltd, Kashiwa, Japan):

(1) $$R2=\frac{1}{T_{2}}=\frac{1}{TE_{2}-TE_{1}}\;ln\left(\frac{S_{1}}{S_{2}}\right)\;$$

where S_1_ and S_2_ are the MR signal intensities at echo times of TE_1_ and TE_2_, respectively.^15^ Thus, R2 is a measure of the dosimeter response. No image noise filters were applied when measuring the values used in this study.

### 2.5 Tolerance

The tolerance for the *R* *2* value comparison was set to 2× the standard deviation of the mean of the dose distribution in the reproducibility test. The tolerance for the dose value comparison was set as a value from dose uncertainty.

## 3 RESULTS

### 3.1 Dose linearity

**FIGURE 4:**
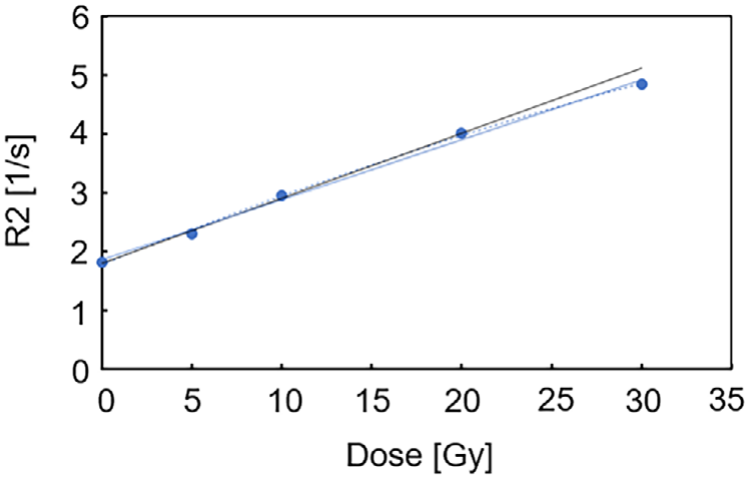
Relationship between dose and *R* *2* value. The solid black line shows the curve fitted against 0–20 Gy doses; the solid blue line shows the curve fitted against 0–30 Gy doses; the blue dotted line shows the curve fitted against 0–30 Gy using a quadratic function. The parameters were: field size 10 × 10 cm^2^, mono‐energy 190 MeV beam with four different target doses (5, 10, 20, and 30 Gy) at 2 cm depth.

The R2 values for doses of 0–20 Gy and 0–30 Gy had determination coefficients of 0.9984 and 0.9955, respectively (Figure 4). The determination coefficient for 0–30 Gy improved to 0.9986 when a quadratic function was used. The irradiated dose distribution did not exceed 20 Gy for most of the following tests, except for dose linearity, dose uncertainty, and dose resolution. Therefore, apart from these three tests, we used the linear *R* *2* ‐ dose curve within the 0–20 Gy range to derive the values.

### 3.2 Dose uncertainty and dose resolution

**FIGURE 5:**
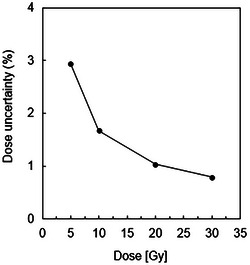
Dose uncertainty of 190 MeV proton beams. The parameters were: field size 10 ×10 cm^2^, 5, 10, 20, and 30 Gy irradiation using 190 MeV at 2 cm depth in the vials.

**FIGURE 6:**
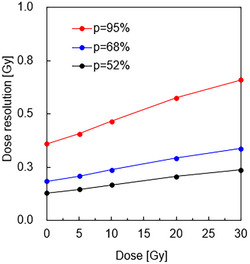
Dose resolution of 190 MeV proton beams. The parameters were: field size 10 × 10 cm^2^, 5, 10, 20, and 30 Gy irradiation using 190 MeV at 2 cm depth in the vials.

Using the results of the dose linearity measurement, the dose uncertainty and dose resolution were measured for all gels for 0–30 Gy, as shown in Figures 5 and 6, respectively. Dose uncertainty^19^ is given by

$$\frac{\sigma_{D}}{D}\left(\%\right)=\sqrt{{\left(\frac{\alpha \sigma_{R_{2}}}{D}\right)}^{2}+{\left(\frac{\sigma_{\alpha }R_{2}}{D}\right)}^{2}+{\left(\frac{\sigma_{D_{0}}}{D}\right)}^{2}}$$

Pixels in the R2 map have values of R2 ± $\sigma_{R2}$. In the calibration graph, the dose is plotted against R2, and linear regression gives the slope (α ± $\sigma_{\alpha \;}$) and intercept ($D_{0}$ ± $\sigma_{D0}$). The standard deviation in R2 can be estimated from the standard deviations in the R2 values of the calibration vials. Dose uncertainty was in the range of 1%–3%.

Dose resolution^20^ is related to dose uncertainty by

$$\;D_{\Delta }^{p}=k_{p}\;\cdot \sqrt{2}\cdot \sigma_{D}$$

where the coverage factor, $k_{p}$, is given by the *t*‐distribution for the appropriate number of experimental degrees of freedom ($k_{95\%}=1.96$). If the confidence level in $D_{\Delta }$ was relaxed to 68%, then the dose resolution would be 0.18–0.34 Gy in the absorbed dose range of 0–30 Gy.

### 3.3 Reproducibility of the dose distribution measurement

**FIGURE 7:**
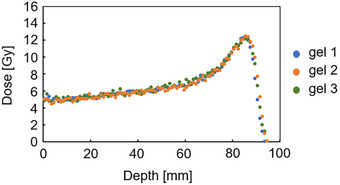
Comparison of PDD curves measured with three gel dosimeters from the same batch of gel. The parameters were: field size 10 × 10 cm^2^, 5 Gy irradiation using 190 MeV at 2 cm depth in the vials. The zero point in the horizontal axis corresponded to 16 mm from the surface of the phantom. PDD, percentage depth dose.

The PDD measurements in the vial made with the three gel dosimeters all showed a range of 80 mm. The doses in plateau and peak regions showed visually pleasing agreement across the three gels (Figure 7). The mean standard deviation of the *R* *2* values ($\sigma_{R2\_m}$) at 10–90 mm depths in the vial was 0.04 across the three gels. The dose difference between gel No. 2 and gel No. 1 was 1.3% ± 4.9% in the plateau region (depth of 0–60 mm), and 1.3% ± 3.2% in the peak region (depth of 61–85 mm). The dose difference between gel No. 3 and gel No. 1 was 1.6% ± 2.8% in the plateau region (depth of 0–60 mm) and 1.6% ± 2.8% in the peak region (depth of 61–85 mm). The dose differences were within the dose uncertainty of 3%, a value that was derived from the results in the dose uncertainty section.

### 3.4 Energy dependence (LET dependence)

#### 3.4.1 TPS comparison

**FIGURE 8:**
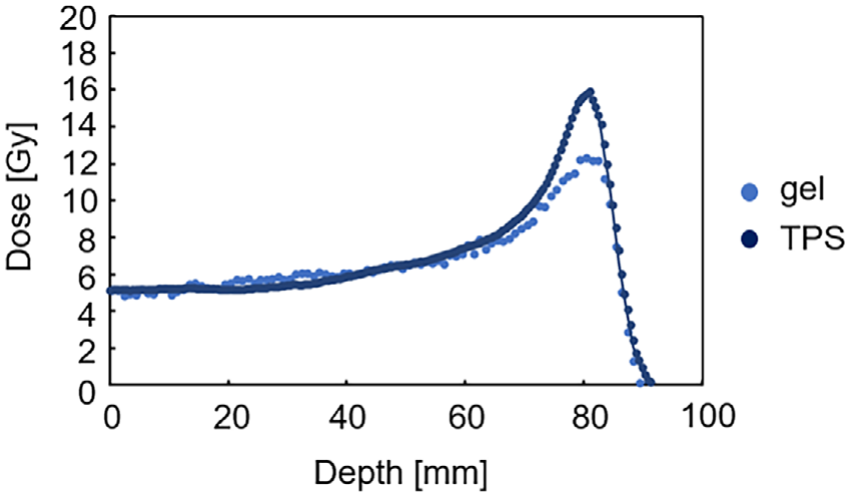
Comparison of PDD curves between the TPS estimates and gel dosimeter measurements. The parameters were: field size 10 ×10 cm^2^, 5 Gy irradiation using 190 MeV at 2 cm depth in the vials. The zero point in the horizontal axis corresponded to 16 mm from the surface of the phantom. PDD, percentage depth dose. TPS, treatment planning system.

The PDD values in the vial were compared between the TPS calculations and the actual measurements. The dose difference among the PDD values was 1.4% ± 4.8% in the plateau region (depth of 0–60 mm) and −12.2% ± 10.4% in the peak region (depth of 61–85 mm) (Figure 8). The dose difference values in the plateau region were within the measurement uncertainty of 3%.

#### 3.4.2 Gel comparison

**FIGURE 9:**
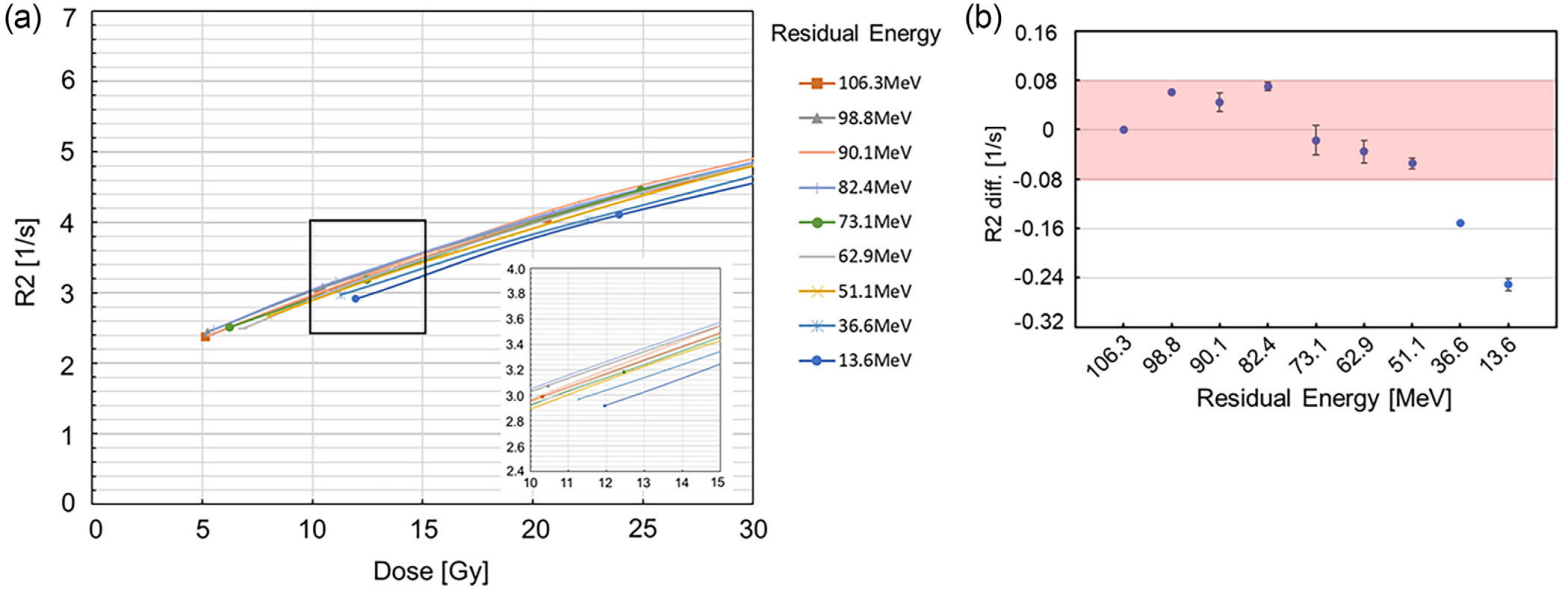
(a) Comparison of measured *R* *2* values at residual energies of 13.6–106.3 MeV and (b) the difference in *R* *2* values of each residual energy of the proton beams at 5–30 Gy. Red region shows 2$\sigma_{R2\_m}$. The parameters were: field size 10 × 10 cm^2^, 5 Gy irradiation using 190 MeV proton beams at 2 cm depth in the vials. Dose values were calculated from the treatment planning system.

The calculated residual energies were 106.3, 98.8, 90.1, 82.4, 73.1, 62.9, 51.1, 36.6, and 13.6 MeV, respectively. For the residual energies, the relationship between the *R* *2* values and the dose values calculated from the TPS is shown in Figure 9a. The difference of *R* *2* values of 51.1–98.8 MeV compared with those of 106.3 MeV were within 2$\sigma_{R2\_m}$, while those of 13.6 and 36.6 MeV exceeded 3$\sigma_{R2\_m}$ in Figure 9b.

### 3.5 Dose rate dependence

**FIGURE 10:**
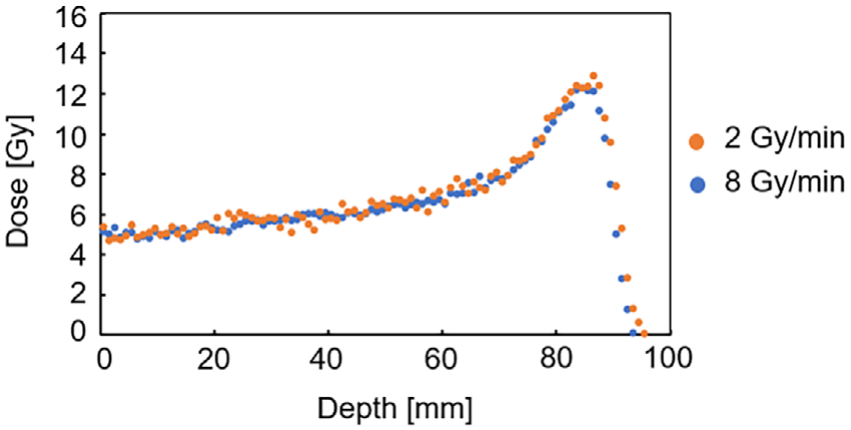
Comparison of PDD curves between 2 and 8 Gy/min dose rates. The parameters were: field size 10 × 10 cm^2^, 5 Gy irradiation using 190 MeV at 2 cm depth in the vials. The zero point in the horizontal axis corresponded to 16 mm from the surface of the phantom. PDD, percentage depth dose.

Dose rate dependence was assessed by observing PDD changes when the dose rate was varied between 2 and 8 Gy/min. The dose difference among the PDD values was 1.8% ± 6.2% in the plateau region (depth of 0–60 mm), and 0.9% ± 2.8% in the peak region (depth of 61–85 mm) (Figure 10). The dose differences in the plateau and peak regions were within the measurement uncertainty.

### 3.6 Beam profile

**FIGURE 11:**
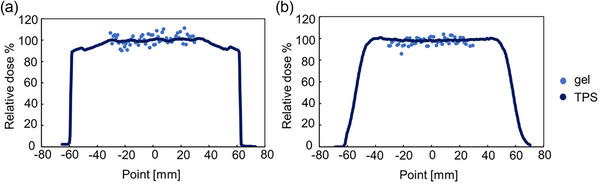
The beam dose profile of the SOBP with a 190 MeV proton beam with two opposite lateral fields: (a) parallel to the beamline, and (b) perpendicular to the beamline. The parameters were: field size 10 ×10 cm^2^, 10 Gy irradiation (5 Gy with each SOBP irradiation), 190 MeV at the isocenter. SOBP, spread‐out Bragg peak.

The dose distributions were scaled using the average dose of the points within ±3 mm of the central axis of the beam profile. The dose profile was measured in a ±2 mm region of the center axis of the proton system (Figure 11). The difference between the TPS and gel‐measured dose in the high dose region (range of −30 to 30 mm) of the SOBP was approximately 1.2% ± 5.2% parallel to the beamline, and 1.3% ± 3.7% perpendicular to the beamline. The differences parallel to the beam and perpendicular to the beam were within the dose uncertainty.

### 3.7 SPR

**FIGURE 12:**
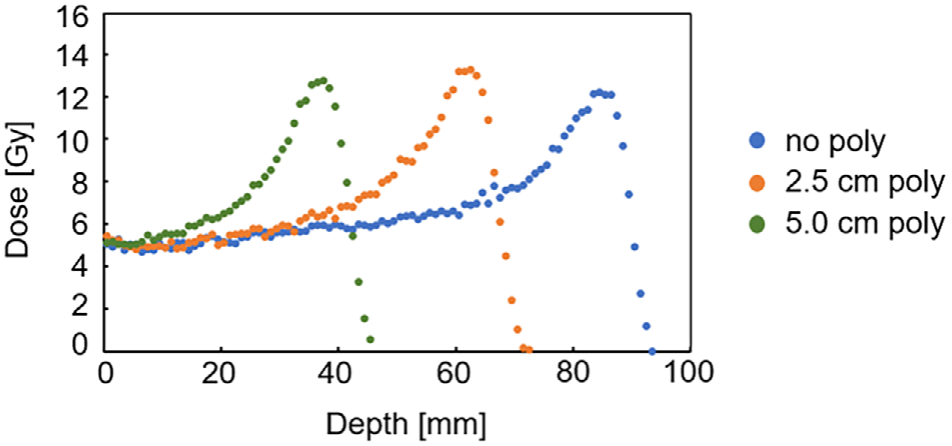
Three PDD curves with and without a polyethylene plate. The zero point in the horizontal axis corresponded to 16 mm from the surface of the phantom. The three conditions were no polyethylene plate, a 2.5 cm polyethylene plate, and a 5.0 cm polyethylene plate. The parameters were: field size 10cm×10cm, 5 Gy irradiation using 190 MeV at 2 cm depth in the vials. PDD, percentage depth dose.

The PDD curves were measured with polyethylene plates of 2.5 and 5.0 cm thickness placed in front of the vial (Figure 12). Subsequently, the SPR was calculated from the two range shifts, $D_{\mathrm{shift}}$, of the difference in the peak location with these polyethylene plates against the area with no polyethylene plate. The calculated SPR value was 1.03.

## 4 DISCUSSION

This study investigated the fundamental characteristics of proton beam measurement using the iVIPET gel dosimeter, including dose linearity, dose uncertainty, dose resolution, reproducibility of dose distribution measurement, LET dependence, energy dependence, dose rate dependence, stopping power ratio, and beam profile. The VIPET dosimeter was found to measure proton beams with high dose reproducibility, regardless of the energy and dose rate. Although some previous reports have examined the fundamental characteristics of proton beam measurement using gel dosimeters, only one previous study evaluated the performance of the VIPET dosimeter, and this was limited to assessing the range and profile. In light of this lack of previously available information, we expect the results obtained in this study to play an essential role in future evaluations of the VIPET dosimeter for proton beams.

Of particular importance is that the iVIPET dosimeter demonstrated high dose linearity in the 0–30 Gy range, with the range measurement results showing that all three measurements were consistent with each other. The dose uncertainties measured in this study were better than or similar to those of other studies (dose uncertainties with three different MRI scanners: 5%–6% at 5–30 Gy,^15^ dose uncertainties in 33 gel batches: 3.3 ± 2.1% at 5 Gy and 4.0 ± 2.5% at 10 Gy).^16^

When the PDD measurements were compared between the TPS‐calculated values and those obtained using the gel dosimeter, a dose reduction of approximately 23% was observed in the peak region of the gel dosimeter. We attribute this dose reduction to the LET dependence of the gel dosimeter, and it is consistent with the reduction levels reported in other studies.^3^, ^12^

When the *R* *2* values were evaluated at various residual energies, no energy dependence was found for 51.1–106.3 MeV, whereas 13.6 and 36.6‐MeV proton beams showed energy dependence. This is an important finding, especially in comparison with photon beam therapy. Specifically, with photon beams the incident energy remains constant throughout the phantom, resulting in no variation in LET. In contrast, the energy of proton beams changes as they travel through the medium. In the case of the 190‐MeV proton beam used in this study, the residual energy varied from 13.6 to 106.3 MeV in the vial. The key characteristic of the VIPET dosimeter, namely, its low energy dependence, was confirmed in this experiment. In the plateau region where the energy ranged from 51.1 to 106.3 MeV, no energy dependence was observed. However, in the high‐LET region near the Bragg peak, energy dependence was evident. This was clearly demonstrated in the comparison between the gel dosimeters as well as in the comparison between the TPS and the gel dosimeters. The dose differences remained within the dose uncertainty even if the dose rate was changed. Therefore, no dose rate dependencies were observed with the iVIPET gel dosimeter.

Baker et al. reported that the BANG gel dosimeter showed LET dependence at the peak region, with the underestimated dose being about 30% at this region.^21^ In addition, Yao et al. reported that energy dependence was not recognized in 80, 100, and 120 MeV incident proton beam irradiation to a NIPAM polymer gel dosimeter.^5^ Therefore, we consider that in comparison with other gel dosimeters, the VIPET gel dosimeter has the advantage of less dose underestimation with comparable energy dependence (LET dependence).

For dose profiles parallel and perpendicular to the beam axis, measurements made near the central axis, where the glass surface effect was minimal, showed differences of approximately 1.2% and 1.3%, respectively, compared with TPS‐calculated values, thereby demonstrating dose uniformity. The beam profile differences in axes horizontal and perpendicular to the beam line were within the dose uncertainty of the gel. The beam profile variations (the standard deviation) in the horizontal and perpendicular axes were approximately 5% and 4%, respectively. The use of a filter is expected to reduce variability in the beam profile, and its application should be considered based on the specific measurement conditions.

Additionally, the measured SPR was 1.03, which matched the estimated SPR value obtained using the CT value of the iVIPET gel (52.62 ± 9.12 HU) and the CT value table in the TPS. These results indicate that while the iVIPET dosimeter exhibits high LET dependence at the peak region, it can measure proton beams without energy or dose rate dependency in range to 2–8 Gy/min, thereby maintaining reproducibility and uniformity in range and dose.

The limitations of this study include the need for more investigations using high‐dose‐rate beams and the image noise filters when obtaining the measured values. In addition, this study was conducted using a single batch of gel dosimeters, and slight variations between batches may result in slight differences in performance. Further studies are required to address these issues.

## 5 CONCLUSION

We clarified the fundamental characteristics of the VIPET dosimeter for proton beam therapy. The VIPET dosimeter maintained high measurement reproducibility and uniformity in range and dose measurements for proton therapy beams without showing dose rate dependence in range to 2–8 Gy/min. The dosimeter exhibited no energy dependence (i.e., LET dependence) in the plateau region. However, caution is required because the influence of high‐LET proton beams in the peak region may contribute to LET dependence comparable to that reported in previous studies.

## AUTHOR CONTRIBUTIONS

Ai Nakaoka collected and analyzed measurement data. Hidenobu Tachibana conceptualized the study, developed the study design, supervised the authors throughout the study, and provided expertise in manuscript preparation. Kenji Hotta collected measurement data. Taeko Matsuura helped to analyze and interpret the data and critically review the manuscript. Yoshihiko Hoshino performed an MRI scan of gel dosimeters.

## CONFLICT OF INTEREST STATEMENT

The authors declare no conflicts of interest.
